# Detecting concept mentions in biomedical text using hidden Markov model: multiple concept types at once or one at a time?

**DOI:** 10.1186/2041-1480-5-3

**Published:** 2014-01-17

**Authors:** Manabu Torii, Kavishwar Wagholikar, Hongfang Liu

**Affiliations:** 1 Department of Radiology, Georgetown University Medical Center, Washington, DC, USA; 2 Department of Health Sciences Research, Mayo Clinic College of Medicine, Rochester, MN, USA

**Keywords:** Natural language processing, Information storage and retrieval, Data mining, Electronic health records

## Abstract

**Background:**

Identifying phrases that refer to particular concept types is a critical step in extracting information from documents. Provided with annotated documents as training data, supervised machine learning can automate this process. When building a machine learning model for this task, the model may be built to detect all types simultaneously (all-types-at-once) or it may be built for one or a few selected types at a time (one-type- or a-few-types-at-a-time). It is of interest to investigate which strategy yields better detection performance.

**Results:**

Hidden Markov models using the different strategies were evaluated on a clinical corpus annotated with three concept types (i2b2/VA corpus) and a biology literature corpus annotated with five concept types (JNLPBA corpus). Ten-fold cross-validation tests were conducted and the experimental results showed that models trained for multiple concept types consistently yielded better performance than those trained for a single concept type. F-scores observed for the former strategies were higher than those observed for the latter by 0.9 to 2.6% on the i2b2/VA corpus and 1.4 to 10.1% on the JNLPBA corpus, depending on the target concept types. Improved boundary detection and reduced type confusion were observed for the all-types-at-once strategy.

**Conclusions:**

The current results suggest that detection of concept phrases could be improved by simultaneously tackling multiple concept types. This also suggests that we should annotate multiple concept types in developing a new corpus for machine learning models. Further investigation is expected to gain insights in the underlying mechanism to achieve good performance when multiple concept types are considered.

## Background

Concept mention detection is the task of identifying phrases in documents that refer to particular concept types. Provided with documents annotated with concept phrases as training data, supervised machine learning can be used to automate concept mention detection. In the biological domain, sets of annotated documents have been developed and made publicly available over the years [1,2]. Similarly in the clinical domain, annotated clinical notes have been recently released to the research community through pioneering efforts [3,4]. These annotated data sets have promoted application of machine learning methods to concept mention detection in the clinical domain [5-8].

When the detection task involves two or more target concept types, there is an option to build one machine learning model for all types (all-types-at-once strategy) or to build multiple models each tackling one type (one-type-at-a-time strategy). The former strategy may have an advantage in exploiting dependency among concept types. In this work, we posed a question if these strategies have impacts on detection performance. We found this question important in two ways. First, it is useful to know if one strategy is better than the other in terms of the detection performance. Second, when a new corpus is developed, the results of the current study may encourage us to annotate additional concept types in order to potentially enhance detection of the target concept type. With current ongoing efforts on corpus development in the clinical domain, we believe this would be a timely question to pose.

In this study, we used two kinds of annotated corpora. The one is a clinical corpus released in the 2010 i2b2/VA natural language processing (NLP) shared-task challenge [4] and the other is a biological literature corpus released in the Joint Workshop on Natural Language Processing in Biomedicine and its Applications (JNLPBA) [9]. The two corpora are different in terms of writing styles as well as concepts presented and annotated, while they share challenges in identifying biomedical concepts, such as difficulty in detecting proper names that may not have initial capital letters and in processing ambiguous acronyms and abbreviations. The best performing system in the i2b2/VA challenge and that in the JNLPBA workshop achieved, respectively, F-scores of 0.852 and 0.726 on the evaluation corpora. These and the other top-ranked systems in the workshops used various machine learning methods, including Hidden Markov Model (HMM), Support Vector Machine (SVM), and Conditional Random Field (CRF), along with various techniques and resources. Our interest in this work is to compare all-type-at-once and one-type- (or a-few-types-) at-a-time strategies, and not to aim for the best performance on these corpora by exploring rich domain features. To focus on this goal, we employed HMM that uses features internal to input text.

## Methods

### Experimental design

One strategy we considered in building a concept detection system was to train one machine learning model that covered all concept types. An alternative strategy tested was to build separate models for different concept types. An HMM program implemented in the LingPipe suite [10] was used to train these models. Detection performance was measured with F-score, the harmonic mean of precision (the number of correctly extracted phrases divided by the number of all extracted phrases) and recall (the number of correctly extracted phrases divided by the number of all phrases to be extracted). We conducted 10-fold cross-validation tests and calculated the average F-score.

### Data

Descriptive statistics of the two data sets used in our experiments are shown in Table 1. The first data set used was a training corpus in the 2010 i2b2/VA NLP shared-task challenge [4]. This data set was made available through our participation in the shared-task challenge and, hence, no additional ethical approval was required for the current study. This corpus consists of 349 clinical documents, including 268 discharged summaries from three institutions and 81 progress notes from one institution. The documents were manually annotated with three concept types: Problem, Test, and Treatment. These annotations (spans of concept phrases) do not overlap each other in text, except for eight annotations that we excluded in the current study.

**Table 1:** Descriptive statistics of the corpora

		**i2b2/VA corpus**		**JNLPBA corpus**
Documents	349		2,000	
Tokens	260,570		492,301	
Concept phrases	Problem	11,968	Protein	30,269
	Test	7,369	DNA	9,530
	Treatment	8,500	Cell Type	6,710
			Cell Line	3,830
			RNA	951

The second data set used was a training corpus of the Bio-Entity Recognition Task in the JNLPBA workshop, which was publicly available online. The corpus consists of 2,000 abstracts of biology research articles retrieved from the MEDLINE database using the search terms (Medical Subject Headings) of ‘human’, ‘blood cells’ and ‘transcription factors’ [9]. It is the same document set as the GENIA version 3.02 corpus, but the thirty six concept types originally annotated in the corpus were simplified to five types for the shared-task workshop: Protein, DNA, Cell Type, Cell Line, and RNA. There is no overlap among annotated concept phrases in this corpus.

### Detection strategies

#### ***One or a few concept types at a time***

In this strategy, independent detection tasks were assumed for subsets of the target concept types. For each subtask, the BIO notation was used [11]. Each token in the corpus was assigned one of the labels, B_*ConceptType*, I_*ConceptType*, and O, representing a token being the Beginning of a concept phrase, Inside of a concept phrase, or Outside of a concept phrase. For example, in order to indicate Problem phrases in the i2b2/VA corpus, the three labels, B_Problem, I_Problem, and O, were used.

#### ***All concept types at once***

In this strategy, a single detection task was assumed for all the target concept types. For example, given the three concept types in the i2b2/VA corpus, one HMM model was built using the seven labels, B_{Problem, Treatment, Test}, I_{Problem, Test, Treatment}, and O.

### Machine learning method

Concept mention detection was often tackled as a sequence labeling problem [4,9]. Input text is viewed as a sequence of tokens and the task is defined as assignment of each token with an appropriate label to demarcate spans of tokens referring to target concept types. We used a sequence labeling program, named CharLmRescoringChunker, from the LingPipe suite [10,12]. This program was chosen because it exploits features internal to text and the performance is not affected by additional external resources and parameters associated with them. Also, this program runs fast and it was desirable in conducting cross-validation tests. A model trained with this program first extracts candidate concept phrases using a first-order Hidden Markov Model (HMM). In HMM, the likelihood of a sequence of labels is calculated based on the two types of probabilities, the transition probabilities and the emission probabilities, learned from the training data set. In the implementation of the LingPipe suite, the emission probabilities that capture the relation between observed words and corresponding labels are calculated using character language models. Transition probabilities that capture the ordering of labels assigned to words are calculated using a bigram model. As for labels to demarcate phrases, instead of using BIO labels given as inputs to the program, enriched BMEWO + representation is used internally [13]. Namely, B of BIO is divided into W (a token of a single-word concept) and B (beginning of a multi-word concept), I into M and E (Middle or End of a multi-word concept), and similarly O into {B, M, E, W}_O, where {B, E, W}_O is further divided based on the type of the neighboring concept. Candidate concept phrases extracted by an HMM model are rescored using another level of character language models to identify the best candidates. We varied the character n-gram size in our experiments, but the experimental results exhibited the same trends across the different choices of the size n and they did not affect our conclusion. Therefore, we chose to report the results for n = 50 that generally yielded good performance. In training the two kinds of models involved, the model for candidate phrase detection and that for their rescoring, eighty and twenty percent of sentences in the training data were used, respectively.

## Results and discussion

**Figure 1:**
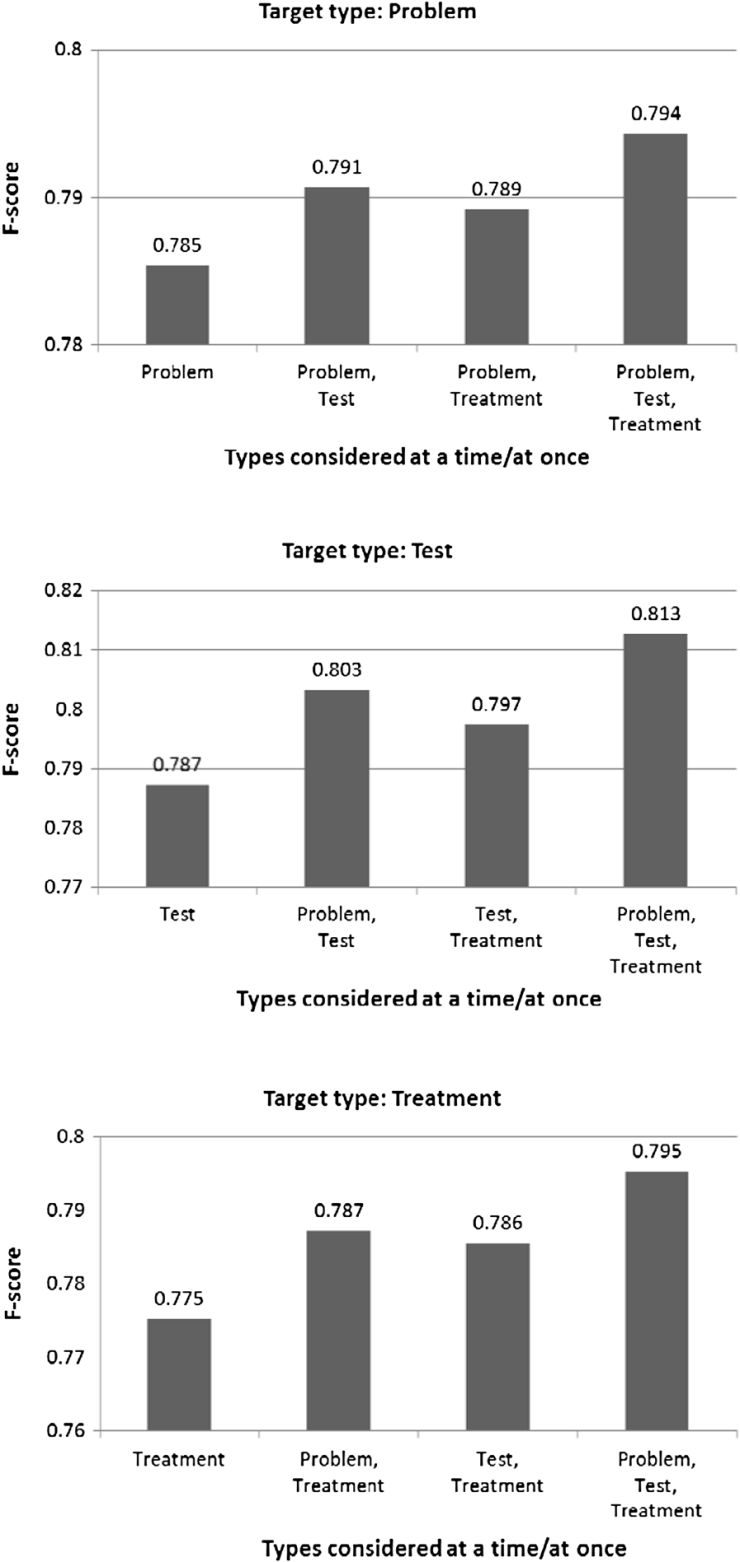
**Detection performance for the 2010 i2b2/VA challenge corpus.** The horizontal axis shows incremental sets of types, including the selected target type (e.g., “Problem” in the top figure), and the rightmost set corresponds to the all-at-once setting. The reported F-scores are for the selected target type.

**Figure 2:**
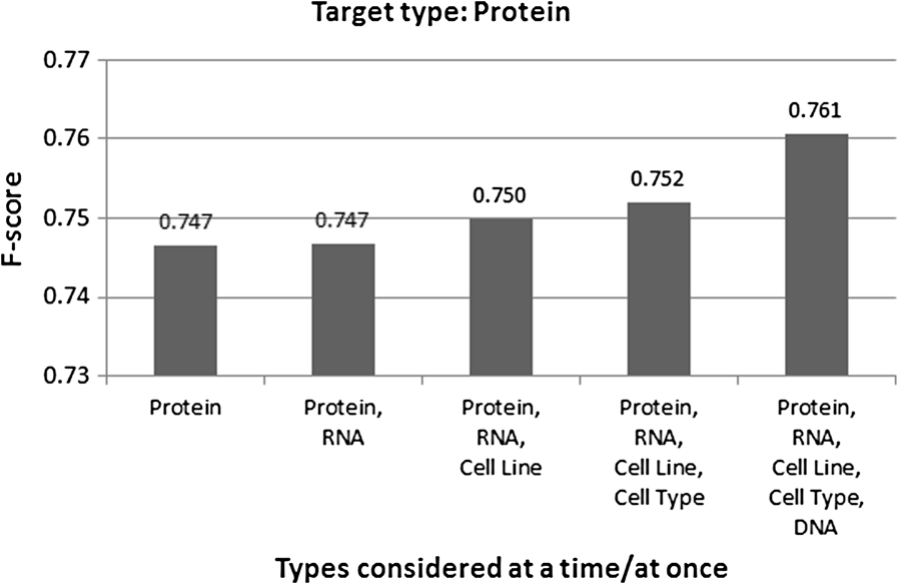
**Detection performance for the JNLPBA corpus.** The horizontal axis shows incremental sets of types, including the selected target type, and the rightmost set corresponds to the all-at-once setting. The reported F-scores are for the selected target type.

Table 2 shows the performance of HMM models trained using the all-types-at-once and the one-type-at-a-time strategies. As stated in the Methods section, we conducted ten-fold cross-validation tests on the two corpora and the detection performance was measured with the average F-score. Figure 1 shows how the detection performance varies when a-few-types-at-a-time was employed for all the three concept types annotated in the i2b2/VA corpus. As for the JNLPBA corpus that is annotated with five concept types, there are many combinations for “a few types” to be selected for the strategy and hence we report on selected combinations for a single target type, Protein, in Figure 2. As seen in the figures as well as in the table, for every concept type annotated in the two corpora, the F-score was the highest when all concept types were considered simultaneously, and the lowest when each type was tackled individually. The differences in the F-scores were statistically significant at the 0.01 alpha level using the two-tailed paired *t*-test. We inspected errors in one-type-at-a-time that were correctly handled in all-types-at-once, anticipating that the latter would take advantage of multiple concept types to identify target phrases. We noticed three major error patterns, and one of them, type confusion, explicitly involves multiple concept types. In the following description of the error patterns, we use examples of the Problem type, but similar instances were observed for the other concept types considered in the experiments.

**Table 2:** Comparison of detection performance

			**TP**	**FP**	**FN**	**Prec.**	**Rec.**	**F-score**
i2b2/VA	Problem	All-at-once	964	267	231	0.783	0.806	0.794
		One-at-a-time	932	244	264	0.792	0.779	0.785
	Test	All-at-once	582	114	153	0.835	0.791	0.813
		One-at-a-time	551	112	185	0.831	0.748	0.787
	Treatment	All-at-once	653	139	196	0.823	0.769	0.795
		One-at-a-time	625	138	223	0.818	0.737	0.775
JNLPBA	Protein	All-at-once	2,373	840	653	0.739	0.784	0.761
		One-at-a-time	2,251	752	775	0.749	0.744	0.747
	DNA	All-at-once	581	270	371	0.683	0.610	0.644
		One-at-a-time	527	339	425	0.609	0.553	0.580
	Cell Type	All-at-once	496	167	174	0.748	0.740	0.744
		One-at-a-time	455	168	215	0.730	0.678	0.703
	Cell Line	All-at-once	233	102	149	0.695	0.610	0.649
		One-at-a-time	212	180	170	0.543	0.554	0.548
	RNA	All-at-once	36	24	59	0.594	0.383	0.462
		One-at-a-time	33	18	62	0.640	0.345	0.447

### Type confusion

In one-type-at-a-time, phrases not of the target type may be falsely detected as target type phrases, e.g., “<Treatment (durg)> for <Treatment (procedure)>” where the latter Treatment phrase was falsely detected as Problem, when Problem alone was tackled.

### Boundary errors

We observed that boundary detection was degraded in one-type-at-a-time. Such cases included simple errors, e.g., “*His melanoma*_Problem_” where the word “His” was missed when Problem type was tackled alone, and also errors involving more complex syntactic patterns, e.g., “*his* <Problem> and <Problem>” where the first Problem phrase (and the word “his”) was missed. Over extension of boundaries was also observed for one-type-at-a-time, but majority of its boundary errors were under extension.

### No detection

Concept phrases correctly identified in all-types-at-once were sometimes totally missed in one-type-at-a-time, e.g., “The patient had no *further complaints*_Problem_” where the Problem phrase was not detected at all when Problem type was tackled alone.

In our review, type confusion was observed less than what we anticipated. For example, when Problem type was tackled alone, across ten folds, there were 42 phrases falsely detected as Problem (false negatives) that were correctly identified as Test (8 phrases) and Treatment (34 phrases) when all the types were tackled simultaneously. Meanwhile, there were 439 Problem phrases that were correctly identified when all the types were tackled but were not identified either partially (199 cases of boundary errors) or fully (240 cases of no detection) when Problem type was tackled alone. Note, however, counting and interpretation of such error types involves subtlety when more closely relevant concept types are densely annotated as in the JNLPBA corpus because boundary errors and type confusion errors coincide frequently. We summarize the numbers of error instances on the i2b2/VA corpus in Table 3. We initially expected that different outputs would be observed among cases involving different concept types, e.g., “<Test> demonstrated <Problem>”, where we might imagine that the recognition of the Test phrase affects that of the Problem phrase or vice versa. We, however, encountered such instances rarely, e.g., “<Test> revealed <Problem>” and “<Test> showed <Problem>”, in which the Problem phrases were not detected when Problem alone was tackled. The detection mechanism in the all-concept-types-at-once strategy needs to be examined to understand the advantage it has.

**Table 3:** Additional errors introduced in one-type-at-a-time on the i2b2/VA corpus

	**Type confusion**	**Boundary error**	**No detection**
Problem	42	199	244
Test	50	92	299
Treatment	47	266	113

In selecting these detection strategies, another important consideration is the time to train and apply detection models. As shown in Table 4, it took more time to train a model using the one-type-at-a-time strategy. Training of an HMM model does not require optimization unlike other popular machine learning methods, such as SVM and CRF, and the increase in the number of target types may not incur extra training time. However, reduction in the training time for all-types-at-once was not expected. That may be attributed to smaller per-type data structures used in all-types-at-once, compared to larger per-type data structures in one-type-at-a-time. The size of the model file was smaller for all-concept-types-at-once, compared to that for one-type-at-a-time, e.g., 159 MB for all-types-at-once and 255 MB for Problem in one run of ten-fold cross-validation.

**Table 4:** **Time to train and apply HMM models on the i2b2/VA and JNLPBA corpora**^**1**^

	**A set of types considered**	**Training (sec)**	**Application (sec)**
i2b2	Problem, Test, Treatment	619	42
	Problem, Treatment	763	41
	Problem, Test	879	42
	Problem	1,117	43
JNLPBA	Protein, DNA, Cell Type, Cell line, RNA	3,010	88
	Protein, DNA, Cell Type, Cell line	3,812	92
	Protein, DNA, Cell Type	4,292	98
	Protein, RNA	4,694	100
	Protein	4,763	98

^1^The experiments were conducted on a server with six-core AMD Opteron 2.8 GHz processors running CentOS 2.6. The reported times are the average of ten runs in ten-fold cross-validation.

Review of individual errors and analysis of run time made us pay attention to the implementation of the HMM program and the impacts of model parameters involved, such as pruning of n-grams in the model and smoothing of probabilities. We explored a wide range of n-gram sizes to test if the choice of the tagging strategy, but it was difficult to explore all the parameters simultaneously, e.g., the n-gram size, the smoothing parameter, and the pruning parameter. Further investigation is required to gain insight in the combination of different parameters, as well as the use of different machine learning paradigms other than HMM.

## Conclusions

In this study, we compared all-types-at-once and one-type-at-a-time strategies in applying HMM taggers on a clinical corpus released in the 2010 i2b2/VA NLP challenge workshop and a biological literature corpus released in the JNLPBA workshop. We also tested a-few-types-at-a-time in building a model. The experimental result shows that tackling multiple concept types at once could improve concept mention detection performance. When building a new corpus, which has become an imminent agenda particularly in the clinical domain, we should consider annotating multiple concept types. The current results are limited to one machine learning method, but notably the best performing systems in the i2b2/VA challenge and the NLPBA workshop employed all-types-at-once for Semi-Markov CRF [14] and HMM with SVM [15]. Further investigation is expected to test various machine learning methods for these different detection strategies.

### Availability of supporting data

The clinical corpus used in this research was a training data set in the Fourth i2b2/VA Shared-Task and Workshop Challenges in Natural Language Processing for Clinical Data. Information of this data set is found at https://www.i2b2.org/NLP/Relations/.

The biology literature corpus used in this research was a training data set for the Bio-Entity Recognition Task in the Joint Workshop on Natural Language Processing in Biomedicine and its Applications. The data set is available at http://www.nactem.ac.uk/tsujii/GENIA/ERtask/report.html.

## Abbreviations

i2b2: Informatics for integrating biology and the bedside; CRF: Conditional random field; FN: False negative; FP: False positive; HMM: Hidden Markov Model; JNLPBA: Joint Workshop on Natural Language Processing in Biomedicine and its Applications; NLP: Natural Language Processing; SVM: Support Vector Machine; TP: True positive.

## Competing interests

The authors declare that they have no competing interests.

## Authors’ contributions

HL conceived the original idea of the study and coordinated the resources for the experiments. MT designed the experiments and carried them out with KW. All authors participated in the analysis of the experimental results. MT drafted the manuscript, and KW and HL significantly revised it. All authors read and approved the final manuscript.
